# Effectiveness of Pamidronate Infusion in the Treatment of Charcot Arthropathy

**DOI:** 10.5704/MOJ.2403.009

**Published:** 2024-03

**Authors:** MY Bajuri, NH Md-Noorpi, MK Yin, I Azman, NS Adib-Adham

**Affiliations:** 1 Department of Orthopaedics and Traumatology, Universiti Kebangsaan Malaysia, Kuala Lumpur, Malaysia; 2 Department of Pharmacy, Universiti Kebangsaan Malaysia, Kuala Lumpur, Malaysia

**Keywords:** charcot, arthropathy, pamidronate, bisphosphonates

## Abstract

**Introduction:**

The objective of this case series is to investigate the efficacy and safety of intravenous infusion of Pamidronate, a second generation bisphosphonate, in the treatment of active Charcot arthropathy.

**Materials and methods:**

All patients with active Charcot arthropathy treated at the medical centre from 1 January 2013 to 30 June 2020 were included in the study. Efficacy outcome was evaluated based on time to consolidate findings observed through radiographic examination, while safety outcome was evaluated based on the incidence of adverse event (AE) occurrence.

**Results:**

A total of 81 patients (37 male, 44 female) diagnosed with active Charcot arthropathy were included. 64.2% of patients were at stage 1 of Charcot arthropathy whereas 35.8% were at stage 2. The mean time to consolidate for stage 1 and stage 2 was 6.50 ± 4.21 months and 3.63 ± 2.92 months respectively (p-value = 0.139). No significant association was observed between gender, ethnicity and disease stage with the consolidation time (p-value >0.05). The rate of AE incidence was 2.5%, observed in 2 patients who developed a fever during the treatment. No other serious AE was observed in the study.

**Conclusion:**

Intravenous Pamidronate infusion is a safe and effective treatment option for Charcot arthropathy.

## Introduction

Charcot arthropathy is a desolation and debilitating complication that occurs in patients with severe neuropathy caused by other medical conditions such as diabetes, tabes dorsalis and syringomyelia. The rate of incidence is about 0.1% - 5.0% in diabetic patients with peripheral neuropathy^1^. Traditional theories concerning the disease pathogenesis of Charcot arthropathy can be divided into two: (1) neurotraumatic theory; and (2) neurovascular theory^1^. The neurotraumatic theory is based on the loss of protective sensation that lead to repetitive trauma and may cause progressive destruction of bone or joint while the patient continued weight bearing. While neurovascular theory involves the possibility of a defect in vasomotor nerves that causes increased blood flow to the extremity. This results in a mismatch between bone synthesis and bone resorption process, which then lead to osteopenia. Patients usually present with the symptoms of swelling, pain, warmth and redness, as a result of the underlying inflammatory process that occurs at the defect site^1^.

Pamidronate is the second generation of bisphosphonates. It is also categorised under nitrogen-containing bisphosphonates which work by inhibiting farnesyl pyrophosphate synthase, promoting agent for the osteoclast attachment to the bone. As a result, the osteoclast was detached from the bone surfaces which lead to the inhibition of bone resorption^2^. Its ability is more potent than etidronate and does not have the narrow therapeutic window exhibited by etidronate in terms of associated bone mineralisation defects and frank osteomalacia^3^. A study showed that Pamidronate is very effective in inhibiting the abnormal osteoclastic and osteoblastic activity that characterises Paget’s bone disease^4^. Although studies had reported that Pamidronate infusion therapy successfully reduced the skin temperature, symptoms, bone turnover and disease activity in patients with Charcot arthropathy, the quality of evidence presented was weak due to the small sample size included for evaluation in the studies^5,6^. Besides, there is a limited study that evaluates the efficacy and safety of Pamidronate on patients with Charcot arthropathy based on consolidation time and incidence of adverse event (AE) occurrence available.

Therefore, this case series was conducted to evaluate the efficacy and safety of Pamidronate on patients diagnosed with active Charcot arthropathy based on time to consolidate through radiographic examination and AE occurrence.

## Materials and Methods

**Fig 1:**
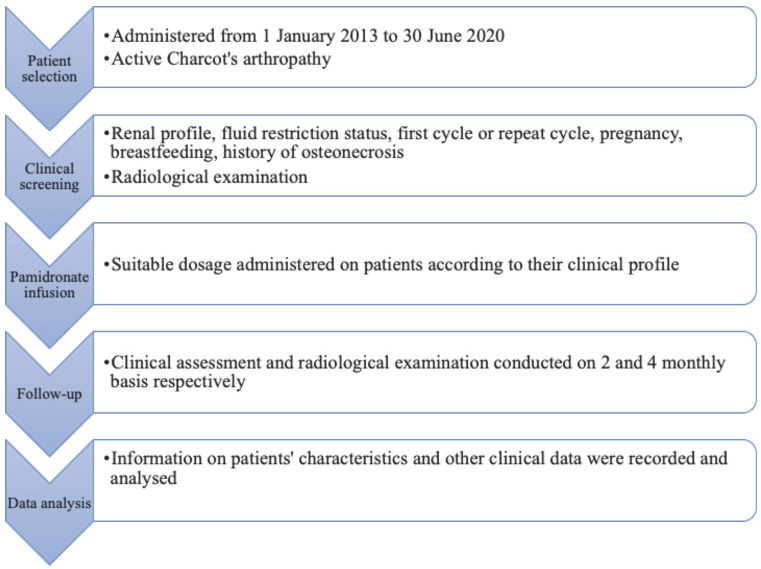
Flow chart of the study.

This is a case series based on data obtained from a retrospective study conducted on patients with active Charcot arthropathy, who were recruited during a period of seven years and six months from 1 January 2013 to 30 June 2020 with an average follow-up period of 24 months. All patients with the diagnosis of active Charcot arthropathy, who were treated at the Foot and Ankle Unit and Diabetic Foot Care Services, Department of Orthopaedics and Traumatology of Hospital Canselor Tuanku Muhriz, were included in this study. The disease stage of Charcot arthropathy was evaluated based on the patient’s clinical and radiological (plain radiograph) examinations conducted on the day of the presentation. Clinical examination was evaluated based on the presence of pain, heat, and swelling with or without erythema, while the radiological examination was evaluated based on radiograph imagery. The disease stage of Charcot arthropathy was then classified according to Eichenholtz Classification shown in (Table I) and recorded. Other demographic-related information such as gender, ethnicity and patients’ comorbidities were also noted. The overall process of the study is represented in (Fig. 1).

**Table I:** Eichenholtz classification.

Disease stage	Significant event	Clinical and radiological observations
Stage 0	-	Joint oedema Radiograph is negative Bone scan may be positive in all stages
Stage 1	Fragmentation	Joint oedema Radiograph shows osseous fragmentation with joint dislocation
Stage 2	Coalescence	Decreased local oedema Radiograph shows coalescence of fragments and absorption of fine bone debris
Stage 3	Reconstruction or consolidation	No local oedema Radiograph shows consolidation and remodeling of fracture fragments

Informed consent was obtained from all patients prior to the treatment session. Before the Pamidronate therapy began, clinical data including renal profile, calcium profile, fluid restriction status, first cycle or repeat cycle, pregnancy, breastfeeding and history of osteonecrosis were recorded since these details would influence the dose of Pamidronate infusion. Patient were given minimum of two doses depend on the stage of patient. Patients in Stage 1 need three doses, while those in Stage 2 require a minimum of two doses. For a patient with a repeat cycle, the date of the previous cycle was checked to ensure that it was at least four months away from the current Pamidronate administration. For the hypocalcemic patient (Ca < 2.10μmol/L), a suitable calcium level was restored with 1g CaCO3 (three times per day) and 0.25μg calcitriol (one time per day) before Pamidronate treatment. A dental examination was performed on a patient with a risk of osteonecrosis. All patients were put on a back slab and advised to perform a non-weight-bearing activity. The patient is advised to utilise either a wheelchair or a non-weight-bearing walking frame for the affected leg.

Injectable Pamidronate [Sandoz Private Ltd, India] was rehydrated in 10ml water for injection diluent and the required dose was syringed out from the vial before being administered to the patients. The volume required for a 60mg dose was 6.7ml while the volume for a 90mg dose was 10ml. A suitable dosage of Pamidronate was administered to the patients according to their renal profile and fluid restriction status: (1) normal renal profile, 90mg in 250ml normal saline run for 2 hours; (2) renal impairment without fluid restriction, 60 – 90mg in 500ml normal saline run for 4 hours; (3) renal impairment with fluid restriction, 60 – 90mg in 250ml normal saline run for 4 hours; (4) Creatinine clearance < 30ml/min or serum creatinine > 265μmol/L, 60mg in 250 to 500ml normal saline, run for 6 hours.

The follow-up protocol on an average two monthly basis was performed on all the patients. Clinical assessment was performed during each visit along with a radiological examination conducted using plain radiograph on the affected foot and ankle in two simple radiologic projection on every four monthly bases. Pre-consolidation radiograph was taken on the day of the presentation. The time to consolidate each patient following Pamidronate treatment was recorded. The data was then analysed using Excel analysis software [Microsoft Corporation, USA]. The demographic data for the was shown in a proportion while time to consolidate (continuous variable) was shown in mean ± standard deviation. Linear regression analysis was performed to determine the risk factors that might influence the time to consolidate in patients. A p-value < 0.05 was considered significant.

## Results

A total of 81 patients diagnosed with active Charcot arthropathy treated at the Foot and Ankle Unit and Diabetic Foot Care Services, Department of Orthopaedics and Traumatology of Hospital Canselor Tuanku Muhriz were included in this study. A total of 45.7% (37) were male while 54.3% (44) were female. The majority of patients were Malay (70.4%), followed by Others (11.1%), Chinese (9.9%) and Indian (8.6%) (Table II). Majority of the patients have underlying disease of diabetes followed by hypertension, dyslipidemia and chronic kidney disease (Table III).

**Table II:** Detailed information of patients.

Patient Characteristics	Number of patients (Percentage %)
Total number of patients	81 (100.0)
Gender	
Male	37 (45.7)
Female	44 (54.3)
Ethnicity	
Malay	57 (70.4)
Chinese	8 (9.9)
Indian	7 (8.6)
Others	9 (11.1)
Disease Stage	
Stage 1	52 (64.2)
Time to consolidate	Mean ± standard deviation (SD) = 6.50 ± 4.21 months
• 2 months	1 (1.9)
• 3 months	7 (13.5)
• 4 months	6 (11.5)
• 5 months	5 (9.6)
• 6 months	13 (25.0)
• 7 months	9 (17.3)
• 8 months	10 (19.2)
• 13 months	1 (1.9)
Stage 2	29 (35.8)
Time to consolidate	Mean ± SD = 3.63 ± 2.92 months
• 2 months	6 (20.7)
• 3 months	8 (27.6)
• 4 months	4 (13.8)
• 5 months	6 (20.7)
• 6 months	2 (6.9)
• 7 months	3 (10.3)
• 8 months	0 (0.0)
• 13 months	1 (1.9)
Overall time to consolidate Adverse event (AE) occurrence	Mean ± SD = 5.20 ± 2.08 months
No	79 (97.5)
Yes (fever)	2 (2.5)

**Table III:** Patients comorbidities.

Patient Characteristics	Number of patients (Percentage %)
Total number of patients	81 (100.0)
Patients’ comorbidities Diabetes Mellitus	66 (81.5)
Hypertension	62 (76.5)
Dyslipidemia	33 (40.7)
Benign Prostatic Hyperplasia	3 (3.7)
Ischemic Heart Disease	11 (13.6)
ESRF	13 (16.0)
Chronic Kidney Disease	20 (24.7)
Cataract	2 (2.5)
Multiple Myeloma	11 (13.6)
Gout	5 (6.1)
Hepatitis C	1 (1.2)
No known medical illness	1 (1.2)
Rheumatoid arthritis	2 (2.5)
Bronchial Asthma	2 (2.5)

**Fig 2:**
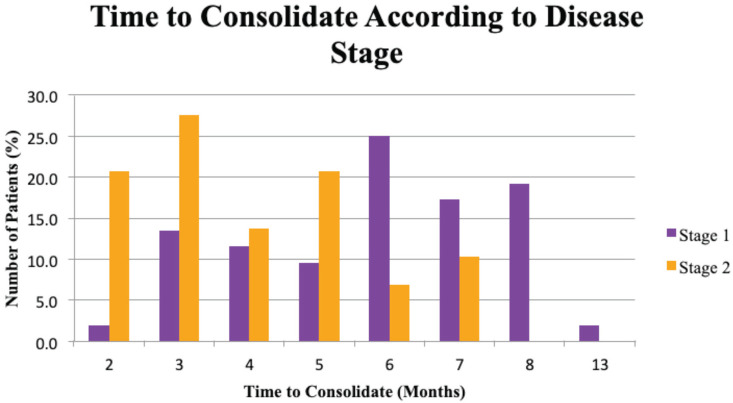
Distribution (%) and time to consolidate (months) of patients according to disease stage.

About two third of the patients which consist of 64.2% were at stage 1 of Charcot arthropathy while the rest were at stage 2. Based on the improvement shown through radiologic examination, the overall mean time to consolidate was 5.20 ± 2.08 months, with the mean time to consolidate for disease stage 1 and stage 2 at 6.50 ± 4.21 months and 3.63 ± 2.92 months respectively (Table II). However, the difference in the mean time to consolidate between stage 1 and stage 2 was not significant (p-value = 0.139). The graph showed a fluctuation in the distribution and time to consolidate patients according to their disease stage (Fig 2).

**Fig 3:**
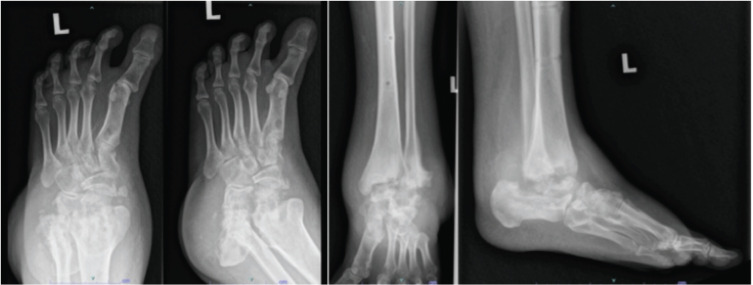
Radiographs image before starting pamidronate infusion treatment.

**Fig 4:**
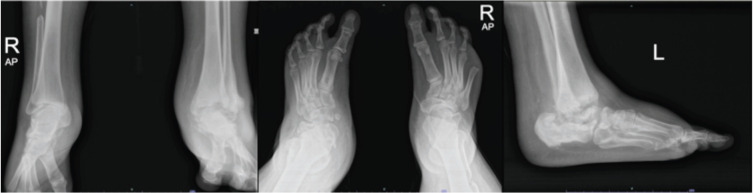
Radiographs image after four months treatment with pamidronate infusion.

The radiograph image showed a structural deformity of the affected ankle and foot from one of the patients (Fig 3). Post consolidation radiograph of the affected foot and ankle showed stabilisation of the radiography changes during the follow-up treatment (Fig 4).

In terms of AE occurrence, only 2.5% (2) developed a fever during treatment whereas the remaining 97.5% (79) did not experience any AE (Table II). However, the reported AEs were not treatment-related. No other serious AE was observed in the study.

The association of each risk factor with the time to consolidate was not significant in which the gender with (p-value = 0.367), the ethnicity with (p-value = 0.267) and disease stage with (p-value = 0.708). It was noted that none of the risk factors below was found to be associated with the time to consolidate.

## Discussion

The results of this study show overall favourable efficacy outcomes of Pamidronate therapy on patients with Charcot arthropathy, which is consistent with previous studies^5-7^. According to the results, the overall mean time to consolidate is 5.20 ± 2.08 months (Table II). This value is similar to that reported by Bharath *et al*, which stated that the mean number of days required for the complete healing process for 45 patients with Charcot arthropathy receiving bisphosphonate treatment was 122 days (4.07 months)^8^. The mean time to consolidate for disease stage 1 was 6.50 ± 4.21 months whereas it was shorter for stage 2 (3.63 ± 2.92 months) although the difference was not statistically significant. This may have happened due to the greater severity of stage 1 compared with stage 2, according to the Eichenholtz classification.

In 1997, Armstrong and his team perfomed a prospective study in which they found that the foot affected by Charcot arthropathy was immobilised after six months. The affected joint loses its reflex to abnormal stress and consequence to joint disintegration which causes the joint to collapse. The injury of the joint triggered the inflammatory reaction, and an increased blood flow localisation was initiated. Other mechanisms like increased histiocytic and osteoclastic activity, removal of blood clots and resorption of avascular bone occurred. The repairing process produced the callus around the fracture’s sites. However, the normal cycle of healing was inefficient due to the overabsorption of the bone which result in osteonecrosis. This process will occur continuously which produces a vicious cycle of injury and repair. The consequences of this disease if left untreated are ulcer formation, deep infection and increased bone destruction^9^. Through oral and intravenous bisphosphonates treatment, the disease can be treated and controlled. A class of drugs since the 1990s, widely indicated for osteoporosis treatment in men and women, is known as bisphosphonates. Their effectiveness depended on their ability to inhibit bone resorption, thus preventing osteoporosis and other related conditions. Generally, the inhibition occurs when bisphosphonate was attached to the hydroxyapatite binding sites on the bone which led to the impairment of the osteoclast to resorb bone^2^.

An observational study involving 219 diabetic patients with acute Charcot arthropathy, who were treated with oral and intravenous bisphosphonates, demonstrated that the resolution time for those treated with bisphosphonates (12 months) was significantly longer than those who were not treated with bisphosphonates (10 months, p-value = 0.005)^10^. This finding is different from the results shown in the current study, which shows that Pamidronate treatment reduces the consolidation time. The observational study theorised that the longer resolution time was probably due to a more severe case of Charcot arthropathy^10^. However, the linear regression analysis of this study shows no significant relationship (p-value > 0.05) between disease stage and consolidation time. Apart from the disease stage, other risk factors such as gender and ethnicity were also found to not influence over the consolidation time. Hence, it is proven that the reduction in consolidation time observed from current study results is solely due to Pamidronate infusion treatment, which further proves the efficacy of Pamidronate infusion.

In terms of the safety outcome of Pamidronate, the incidence of AE occurrence was quite low according to the results. Only 2 patients (2.5%) developed a fever following Pamidronate infusion while the remaining 79 patients (97.5%) did not suffer from any complications during the treatment. The two cases of fever were also found to be unrelated to Pamidronate treatment. The rate of AE incidence reported in the current study (2.5%), which involves a larger sample size of 81 patients, is much lower than that reported by Anderson *et al*^11^, which involves a smaller sample size of 33 patients. Anderson *et al* reported a staggering 60% of patients treated with Pamidronate infusion developed transient fever following the treatment, suggesting a pro-inflammatory effect caused by Pamidronate infusion^11^. However, the low incidence rate of AE from the current study which covers a larger sample size may provide strong evidence to prove that Pamidronate is safe and does not cause any other serious complications in patients with Charcot arthropathy. Treatment of Charcot's joints remains difficult, and involves prolonged periods without weightbearing, immobilisation, and surgical salvage procedures to avoid amputation. The pathogenesis is multifactorial; many studies have demonstrated the central role of inflammation and the Receptor Activator of NF-κB ligand (RANKL)-Receptor Activator of NF-κB (RANK)-Osteoprotegerin (OPG) pathway in the acute phase of the disease, resulting in the serum overexpression of RANKL. This overexpression and activation of this signal lead to increased osteoclast activity and osteolysis, which is a prelude to bone destruction. Drugs that act at different levels in this pathway are anti-RANKL monoclonal antibodies (Denosumab), bisphosphonates (BP), and calcitonin^12,13^.

There are several limitations present in this study. One of them is the uneven distribution of patients according to ethnicity and disease stage. Such uneven distribution may result in bias during statistical analysis. Another one is the parameter used to evaluate the efficacy of Pamidronate. Only one parameter – consolidation time based on radiographic examination, was included for efficacy evaluation. The exclusion of other important parameters like bone turnover markers, skin temperature, symptoms and pain score in the study fails to present a more detailed therapeutic response of Pamidronate in patients with Charcot arthropathy. Besides, the risk of recall bias was found in this study as the clinical assessment was performed subjectively. Lastly, the lack of a control group in this study can become a research gap to perform case-control studies for future research.

## Conclusion

Intravenous Pamidronate infusion is a safe and effective treatment option for patients with Charcot arthropathy as it reduces the time to consolidate and does not cause any serious complications to the patients. However, further studies covering more parameters are needed to present the therapeutic response of Pamidronate in a more detailed manner.
